# eXCube2: Explainable Brain-Inspired Spiking Neural Network Framework for Emotion Recognition from Audio, Visual and Multimodal Audio–Visual Data

**DOI:** 10.3390/biomimetics11030208

**Published:** 2026-03-14

**Authors:** N. K. Kasabov, A. Yang, Z. Wang, I. Abouhassan, A. Kassabova, T. Lappas

**Affiliations:** 1 Knowledge Engineering and Discovery Research Institute (KEDRI), School of Engineering, Computer and Mathematical Sciences, Auckland University of Technology (AUT), WZ Building, St. Paul Street, Auckland 1010, New Zealand; zhaoxin.wang@autuni.ac.nz; 2 Institute for Information and Communication Technologies (IICT), Bulgarian Academy of Sciences, Acad. Georgi Bonchev Str., Bl. 2,25A, 1113 Sofia, Bulgaria; 3 KEC Ltd., 47C Nihill Cr., Auckland 1071, New Zealand; iabouhassan@tu-sofia.bg (I.A.); assia.k@protonmail.com (A.K.); 4 ManaBridge Ltd., Queenstown 9371, New Zealand; alexanderyanghx@gmail.com; 5 Department of Industrial Applications of Computers, Technical University of Sofia, ul. “Professor Georgi Bradistilov” 11, 1756 Sofia, Bulgaria; 6 Department of Marketing and Communication, School of Business Administration, Athens University of Economics and Business, 28is Oktovriou 76, 104 34 Athina, Greece; ted@aueb.gr

**Keywords:** biomimetic systems, brain-inspired computation, spiking neural networks, emotion recognition, NeuCube

## Abstract

This paper introduces a biomimetic framework and novel brain-inspired AI (BIAI) models based on spiking neural networks (SNNs) for emotional state recognition from audio (speech), visual (face), and integrated multimodal audio–visual data. The developed framework, named eXCube2, uses a three-dimensional SNN architecture NeuCube that is spatially structured according to a human brain template. The BIAI models developed in eXCube2 are trainable on spatio- and spectro-temporal data using brain-inspired learning rules. Such models are explainable in terms of revealing patterns in data and are adaptable to new data. The eXCube2 models are implemented as software systems and tested on speech and video data of subjects expressing emotional states. The use of a brain template for the SNN structure enables brain-inspired tonotopic and stereo mapping of audio inputs, topographic mapping of visual data, and the combined use of both modalities. This novel approach brings AI-based emotional state recognition closer to human perception, provides a better explainability and adaptability than existing AI systems. It also results in a higher or competitive accuracy, even though this was not the main goal here. This is demonstrated through experiments on benchmark datasets, achieving classification accuracy above 80% on single-modality data and 88.9% when multimodal audio–visual data are used, and a “don’t know” output is introduced. The paper further discusses possible applications of the proposed eXCube2 framework to other audio, visual, and audio–visual data for solving challenging problems, such as recognizing emotional states of people from different origins; brain state diagnosis (e.g., Parkinson’s disease, Alzheimer’s disease, ADHD, dementia); measuring response to treatment over time; evaluating satisfaction responses from online clients; cognitive robotics; human–robot interaction; chatbots; and interactive computer games. The SNN-based implementation of BIAI also enables the use of neuromorphic chips and platforms, leading to reduced power consumption, smaller device size, higher performance accuracy, and improved adaptability and explainability. This research shows a step toward building brain-inspired AI systems.

## 1. Introduction: Toward Brain-Inspired Biomimetic Systems for Audio, Visual and Audio–Visual Pattern Recognition

### 1.1. Problem Definition

Current technologies for speech recognition and face recognition have advanced significantly in recent years, driven by modern statistical and neural network methods [1,2,3,4,5,6,7,8,9,10,11]. However, voice and face data can be used to address many other challenging AI problems [12,13,14,15,16]. An open problem is the development of AI systems that use voice and vision data to recognize and explain human brain states, such as emotional states and brain diseases. Current voice and computer vision technologies need to be further developed, and new approaches must be created to make AI systems closer to human perception, human expression, and human understanding, and perhaps even human consciousness [14]. One way to target this goal is to develop brain-inspired AI systems (BIAI).

Current brain-inspired systems are mostly based on spiking neural networks (SNNs) [9,17]. An example is the brain-inspired SNN architecture NeuCube, introduced in [18].

The aim of the proposed novel eXCube2 SNN framework is to recognize emotional states from audio, visual, and multimodal audio–visual data using for the first time a brain-inspired approach. While based on the NeuCube architecture, the eXCube2 framework is a novel one that introduces new methods for the problem in hand.

### 1.2. Related Work

#### 1.2.1. Audio-Based Emotional State Recognition

Recognising emotions from speech has been approached using both classical machine learning and deep learning. Traditional methods extract acoustic descriptors such as pitch, energy, and spectral shape from the speech signal and classify them using support vector machines (SVM) or similar classifiers. On the RAVDESS dataset, this ap-proach achieves approximately 62.48% unweighted accuracy for three class (low/neutral/high) arousal detection [19,20], establishing a conventional baseline.

Deep learning methods improve on this by learning features directly from the audio. Issa et al. [21] trained a convolutional neural network (CNN) on multiple audio representations, achieving 71.61% on RAVDESS across 8 emotion classes under speaker-independent evaluation. Mustaqeem and Kwon [22] combined convolutional and recurrent layers to capture both short-term and long-term temporal patterns, reaching 80%.

A further step came with large pre-trained speech models such as wav2vec 2.0 and HuBERT, which learn general speech representations from thousands of hours of unlabelled recordings before being applied to emotion recognition. Pepino et al. [23] showed that wav2vec 2.0 features achieve 84.1 ± 1.2% on RAVDESS (8 classes, 5-fold cross-validation). These models require substantial computational resources for pre-training, and their accuracy can degrade significantly under strict speaker-independent evaluation conditions [24]. They are not incrementally adaptable to new accents and pronunciations.

#### 1.2.2. Visual and Multimodal Emotion Recognition

Facial expression recognition typically relies on geometric features such as facial landmark positions and movements, or on visual features learned by CNNs from face images. Combining audio and visual information through multimodal fusion generally improves accuracy, as the two signals provide complementary cues about emotional state.

On RAVDESS, Luna-Jiménez et al. [25] evaluated a multimodal system combining a pre-trained audio CNN with a recurrent network for facial features, achieving 80.08% on 8-class emotion recognition under speaker-independent 5-fold cross-validation. Audio alone reached 76.58% and face alone 57.08%. In a follow-up study [26], replacing the audio component with a fine-tuned wav2vec 2.0 model and incorporating facial action units improved multimodal accuracy to 86.70% under the same protocol.

#### 1.2.3. Spiking Neural Networks for Emotion Recognition

Spiking neural networks (SNNs) offer a biologically grounded alternative to conventional deep learning, representing information through discrete temporal events rather than continuous activations. Despite their natural suitability for processing temporal signals, relatively few studies have applied SNNs to emotion recognition. Mansouri-Benssassi and Ye [27] evaluated SNNs for both facial and speech emotion recognition on RAVDESS, finding that SNNs maintained significantly higher accuracy than CNNs and SVMs under noisy conditions, demonstrating greater robustness to real-world signal degradation. Wysoski et al. [9] presented an earlier evolving SNN framework for audio–visual processing, followed by the use of NeuCube SNN [28]. Transformer-inspired SNN architectures for multimodal classification have also been explored [29], reflecting growing interest in neuromorphic approaches for affective computing. All these used traditional feedforward SNN and not a brain-inspired SNN architecture.

#### 1.2.4. Transformer-Based Audio–Visual Models

Most of the Transformer-based models are directed to speech recognition, speaker separation, e.g., [30,31], which is a different task from emotional state recognition, still their models are worth analysing as they deal with audio–visual data processing.

MMST proposes a Multimodal Sparse Transformer Network for noise-robust audio–visual speech recognition (AVSR) by strengthening motion-aware visual modelling and improving attention selectivity over long input sequences [30]. The framework aligns and models three streams—audio (A), lip appearance (V), and lip motion (O)—where motion is explicitly captured via optical flow and encoded with a spatiotemporal front-end. To better inject dynamic articulatory cues into the visual representation, the authors introduce Cross-Modal Attention Fusion (CMAF), which uses visual features as queries and motion features as keys/values to produce an enhanced visual embedding for decoding. In addition, MMST adopts a top-q sparse attention strategy within the Transformer, retaining only the most relevant attention positions for each query to suppress irrelevant context and improve robustness in long-range temporal modelling. Experiments on standard AVSR benchmarks (including LRW for word-level pretraining and LRS2/LRS3 for sentence-level evaluation) show that both sparsification and motion-aware fusion contribute to a consistent WER improvements. On LRS2, introducing sparse attention yields an approximately 1.6% absolute WER reduction compared with a baseline such as TM-seq2seq, while incorporating the motion stream with CMAF provides a further ~1.4% absolute WER reduction over simple concatenation-based fusion; the gains are reported to be especially evident under low-SNR noise conditions, indicating enhanced robustness in adverse acoustic environments [30].

#### 1.2.5. LSTM Models for Audio–Visual Data Processing

Most of the LSTM models are directed to speech recognition, e.g., [32,33], which is a different task from emotional state recognition; nevertheless, their models are worth analysing as they deal with both speech and image in their integration.

Reference [32] addresses audio–visual speech recognition (AVSR) by proposing a multimodal recurrent neural network (multimodal RNN) that jointly models the temporal/sequential structure of both audio and visual streams, unlike prior deep AVSR approaches that typically ignore the sequential nature of one modality. The model consists of three parts: (1) an audio encoder based on (uni-/bi-)LSTM; (2) a visual encoder that uses a CNN on mouth-region frames followed by (uni-/bi-)LSTM to capture visual dynamics; and (3) a fusion module that combines the two modalities using a learned multimodal layer built on weighted state summaries of each stream [32]. Experiments are conducted on the benchmark AVletters dataset: 10 speakers (5 male, 5 female), each pronouncing isolated letters A–Z with 3 repetitions, totalling 780 utterances. Visual input is the mouth ROI at 60 × 80 pixels, with 23–79 video frames per utterance; audio is represented using 26-dimensional MFCC features, with 12–40 MFCC frames per utterance. For fusion strategies, the multimodal-layer fusion is reported as the strongest in most SNR conditions and is used as the main configuration thereafter. In direct comparison against the classical AVletters approach by Matthews et al., the proposed multimodal RNN achieves higher best accuracies across all noise levels, with particularly large gains under severe noise: 87.7% (clean) and 70.0% (0 dB) versus 86% (clean) and 42% (0 dB) for Matthews et al. [32]. These results indicate that explicitly modelling both modalities as sequences and learning a trainable fusion layer provides substantial robustness benefits when the acoustic channel is degraded.

Reference [33] studies audio–visual speech recognition (AVSR) using a high-frame-rate 3D audio–visual corpus and proposes a framework that remains useful even when visual input is unavailable at test time. The authors argue that most prior AVSR work relies on 2D corpora with relatively low video sampling rates, whereas their 3D facial motion capture provides visual features at up to 100 Hz and enables direct extraction of discriminative articulatory motion cues. Methodologically, they introduce a visual feature-generation-based bimodal CNN: an inversion LSTM-RNN is trained to predict visual features from audio, and a bimodal CNN-HMM integrates audio with either (i) ground-truth visual features or (ii) LSTM-generated visual features, thereby eliminating the strict requirement for true visual modality at inference time [33]. Experiments use a multi-speaker Mandarin Chinese 3D audio–visual corpus containing 28 speakers (14 female, 14 male) and designed with seen/unseen speaker splits in development and test sets to assess generalisation.

Reference [34] presents a hybrid deep-learning approach for speech emotion recognition (SER) that integrates 1D convolutional neural networks (CNNs) with stacked Residual Bidirectional LSTMs (RBi-LSTMs) to jointly model local spectro-temporal patterns and longer-range temporal dependencies in emotional speech. The method uses a holistic multi-feature representation, combining MFCCs (with derivatives), Chroma, Mel-spectrogram features, Zero-Crossing Rate (ZCR), and RMS energy; these per-frame descriptors are concatenated into a 260-dimensional feature vector and padded to a fixed sequence length of 130-time steps to form a consistent model input of shape (130, 260) [34]. The evaluation is conducted on a combined dataset created by merging two standard SER corpora: RAVDESS and TESS, with the goal of improving variability exposure and generalization. The merged dataset is split into 70% training (3976 samples), 15% validation (852 samples), and 15% held-out test (852 samples); the task covers eight emotion classes [34]. On the held-out test set, the proposed CNN–RBiLSTM model achieves 96.83% test accuracy with test loss = 0.1324. The class-wise report indicates strong and relatively balanced performance, with macro/weighted averages around 0.96–0.97 and the main confusions concentrated in comparatively weaker classes such as calm (lower precision) and sad (lower recall). The authors attribute the overall performance to (i) the complementary information captured by the multi-feature input and (ii) improved temporal modelling and training stability provided by residual connections in the bidirectional LSTM stack [34].

### 1.3. Positioning of Our Present Work

Several gaps remain in the current literature. Explainability is rarely addressed, with most deep learning models offering little insight into the spatio-temporal patterns they have learned and into adaptability to new data. While brain-template-structured SNNs have been applied to emotion recognition from neuroimaging data such as EEG [17,18], no prior work has combined tonotopic and topographic input mappings within a brain-template SNN for emotional state recognition from audio, visual, and multimodal audio–visual data. The eXCube2 framework addresses these gaps by combining a brain-template-structured 3D SNN with biologically motivated input mappings, STDP-based learning, and interpretable connectivity patterns, remaining suitable for neuromorphic hardware deployment. In this sense, this is the first attempt to show that brain-inspired SNN architectures can capture and explain emotional states from audio, visual and audio–visual data, achieving also better or competitive accuracy to the other methods.

## 2. Methods: A General eXCube2 Framework and Models for Emotional State Recognition Based on Audio-, Visual and Multimodal Audio–Visual Data

### 2.1. Why Use Brain-Inspired SNN and the NeuCube Architecture for Audio–Visual Data?

Spiking neural networks (SNNs) are biologically inspired artificial neural networks in which information is represented as binary events (spikes), similar to action potentials in the brain, and learning is also inspired by principles observed in the brain. SNNs are also universal computational mechanisms [17]. Learning in SNNs refers to changes in the connection weights in the network. Many learning paradigms, such as Spike-Timing-Dependent Plasticity (STDP), are inspired by the Hebbian learning principle. In STDP, synaptic weights are adjusted based on the temporal order of the incoming spike (pre-synaptic) and the output spike (post-synaptic). This synaptic weight adjustment determines synaptic potentiation, known as long-term potentiation (LTP), when the synaptic weight increases (positive change). On the other hand, synaptic depression, known as long-term depression (LTD), occurs when the synaptic weight decreases (negative change). If a pre-synaptic spike arrives before (after) a post-synaptic spike, the synaptic link between the two neurons is potentiated (depressed). Thus, learning in the network depends on spike times, which leads to changes in synaptic strength.

STDP is defined mathematically in Equation (1):

(1) $$W\;\left(t_{pre}-t_{post}\right)=\left\{\begin{array}{c} {\;A}^{+}\;e^{(t_{pre}-t_{post})/\tau_{+}},\;\;\;\;\;\;{\mathrm{if}\;t}_{\mathrm{pre}}{\;<\;t}_{\mathrm{post}}\;\\ -A^{-}\;e^{(t_{post}-t_{pre})/\tau_{-}}\;\;\;\;\;{\mathrm{if}\;t}_{\mathrm{pre}}{\;>\;t}_{\mathrm{post}} \end{array}\right.$$

where $W\;\left(t_{pre}-t_{post}\right)$ is the change in weight as a function of the difference between the pre- and post-synaptic spike times, τ+ and τ− are the LTP and LTD time constants, respectively, and $A^{+}$ and $A^{-}$ are the maximum adjustment to synaptic weight when $t_{pre}-t_{post}$ approaches zero.

Overall, an SNN trained with the STDP rule can capture spatio- and spectro-temporal patterns from data, where input neurons are spatially distributed, and connection weights learn temporal associations between them.

Izhikevich [35] has shown that similar activation patterns (called ‘polychronous waves’) can be generated in an SNN reservoir with recurrent connections to represent short-term memory. This is a further extension of the ‘synfire chain’ theory by Abeles [36]. The above principles are utilized in [37,38,39,40] for the creation of spatio-temporal associative memories in SNN, which is a brain-inspired principle in audio–visual perception [41].

**Figure 1:**
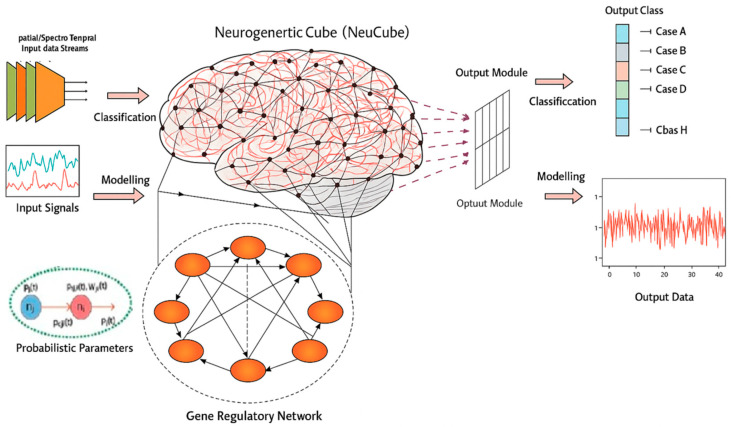
The NeuCube architecture (adapted from [18]).

The eXCube2 architecture is based on the NeuCube SNN brain-inspired architecture (Figure 1) [18,42].

The functionality of the NeuCube architecture is described as follows [18]:

Temporal inputs (features) are converted into spike trains.

Inputs are mapped spatially into a 3D SNNcube that consists of spiking neurons spatially organized in a topological 3D map. For modelling cognitive brain-related data, the SNNcube is built using a brain template, such as MNI, etc. (e.g., [43,44,45,46]).

An output classifier/regressor SNN is connected to neurons from the SNNcube, e.g., deSNN [47].

The SNNcube structure is initialized as a small world connectivity 3D structure of spiking neurons.

Unsupervised learning is performed in the SNNcube using STDP.

Supervised learning is performed in the output SNN module, e.g., deSNN for classification.

The learned connectivity patterns in the SNNcube can be interpreted as deep knowledge, representing deep spatio-temporal patterns in the data. Learned connectivity patterns in the deSNN output module can be interpreted for rule extraction related to outputs [48,49].

The model is further trained and adapted using new data, during which connections are modulated within the SNNcube and additional output neurons are generated in the deSNN classifier to capture emerging patterns and previously unseen classes.

### 2.2. The General eXCube2 Framework

**Figure 2:**
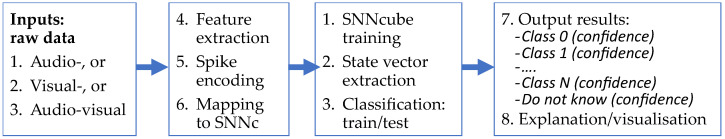
The problem of detecting an emotional state from speech, image, or from both modalities, is represented as a classification problem.

The problem of detecting an emotional state using audio, visual, or both modalities is represented here as a classification problem (Figure 2).

**Figure 3:**
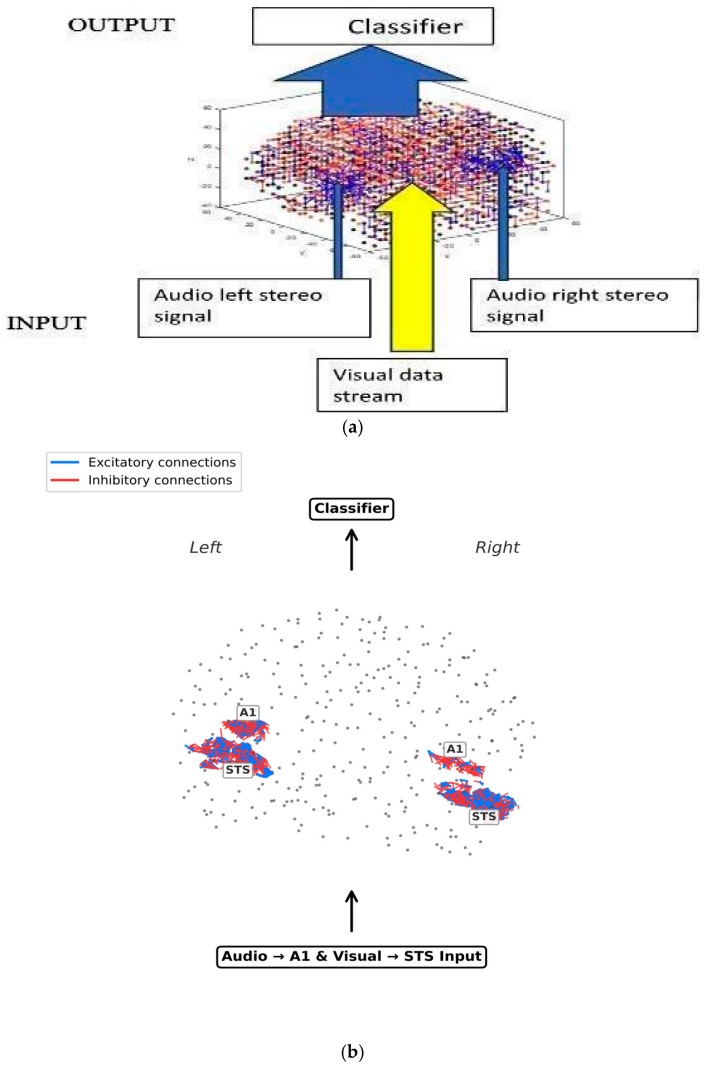
(**a**) The eXCube2 architecture using a brain template for the SNNcube, tonotopic mapping of audio signals and topographic mapping of images for the realisation of the functional diagram from Figure 2. (**b**) Excitatory (blue) and inhibitory (red) connections in the auditory A1 and visual STS areas are shown. Inputs enter this areas bilaterally. A state vector computed from the neuronal activity is classified (A1 = primary auditory cortex; STS = superior temporal sulcus).

The eXCube2 architecture applies brain-inspired tonotopic mapping of audio signals and topographic (retinotopic) mapping of images into the 3D SNNcube, and the learned or recalled patterns in the SNNcube are then classified (Figure 3a,b).

### 2.3. Experimental Data

For the initial development and testing of the eXCube2, we use part of the Ryerson Audio–Visual Database of Emotional Speech and Song (RAVDESS): a dynamic, multimodal set of facial and vocal expressions in North American English [50,51]. The dataset is available at https://zenodo.org/records/1188976 (accessed 30 November 2025), along with https://zenodo.org/records/3255102 (accessed 1 December 2025). Examples of the data are available at https://www.youtube.com/watch?v=cxMK2J0P7J0 (accessed 30 November 25)

The Ryerson Audio–Visual Database of Emotional Speech and Song (RAVDESS) contains 7356 files (total size: 24.8 GB). The dataset includes 24 professional actors (12 female and 12 male) vocalizing two lexically matched statements in a neutral North American accent. Speech includes calm, happy, sad, angry, fearful, surprised, and disgust expressions, and song contains calm, happy, sad, angry, and fearful emotions. Each expression is produced at two levels of emotional intensity (normal, strong), with an additional neutral expression. All conditions are available in three modality formats: audio-only (16-bit, 48 kHz, .wav format), audio–video (720 p H.264, AAC 48 kHz, .mp4 format), and video-only (no sound). The RAVDESS was developed by Dr Steven R. Livingstone [51].

For the experimental study, the following labelling of the data has been used:

- Class 0 = Low arousal: neutral, calm, sad;
- Class 1 = High arousal: happy, angry, fearful, disgust, surprised.

We adopt binary arousal classification (high vs. low) rather than the full 8-class categorization for several reasons. First, arousal is a well-established dimension of emotional state, and the RAVDESS emotions naturally partition along this axis. Second binary arousal detection is directly applicable to real-world screening, such as patient monitoring or confrontation detection, and can serve as a first-pass filter before finer-grained assessment.

### 2.4. Audio Feature Extraction and Feature Encoding in eXCube2

Different features can be extracted from raw sound data and used for different applications. In the context of brain state recognition, this paper uses mel-spectrogram features, after considering and comparing them with three other possible feature types, as shown in Table 1.

Each feature is mapped into the 3D SNN as an input neuron. Table 1 presents the audio features investigated in this study.

**Figure 4:**
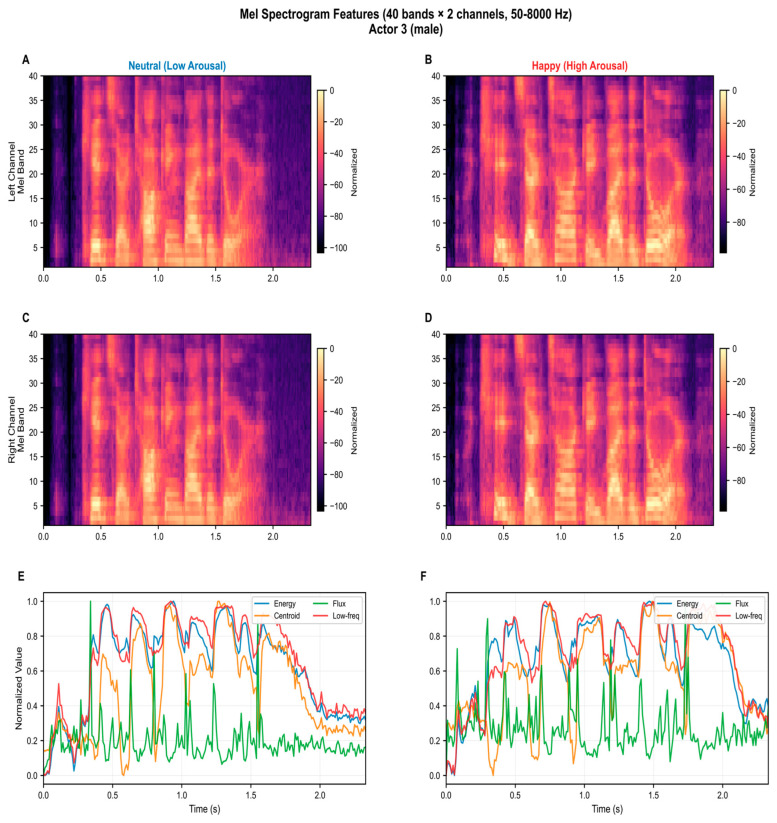
Examples of extracted mel-spectrogram features from neutral speech (**left**) and aroused/happy speech (**right**) ((**A**,**B**) show data from the left channels; (**C**,**D**) show data from the right channels). (**E**,**F**) show features of the voice, such as: RMS energy reflects the instantaneous loudness of the utterance; spectral centroid captures the brightness of the voice, defined as the amplitude-weighted mean frequency of the spectrum; spectral flux measures the rate of spectral change between consecutive frames, indicating articulatory dynamics; low-frequency energy (0–345 Hz) represents activity in the fundamental frequency and lower harmonic regions associated with vocal pitch. Together, these features characterise the prosodic and timbral properties known to differentiate emotional expression in speech [20,52].

The audio features are mapped into the SNNcube as input neurons to both the left and right areas of the SNNcube, which correspond to the left and right auditory cortex according to the selected brain template. Each of the above features can be used in the development and implementation of an eXCube2 model for specific applications. Mel-spectrogram features, as used in the current implementation of eXCube2, are shown in Figure 4.

**Figure 5:**
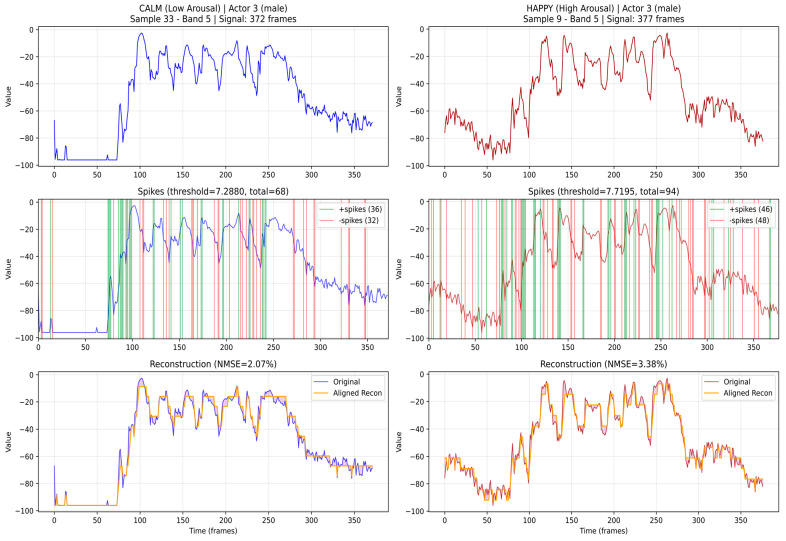
Spike encoding of the mel-spectrogram audio features from Figure 4 (**top**) using the Step-Forward method (**middle**) (see [17]) and reconstructing the signals from the spikes back to the original ones (**bottom**). It shows that the used encoding method is suitable for the selected features as it results in a small error after reconstruction.

**Figure A1:**
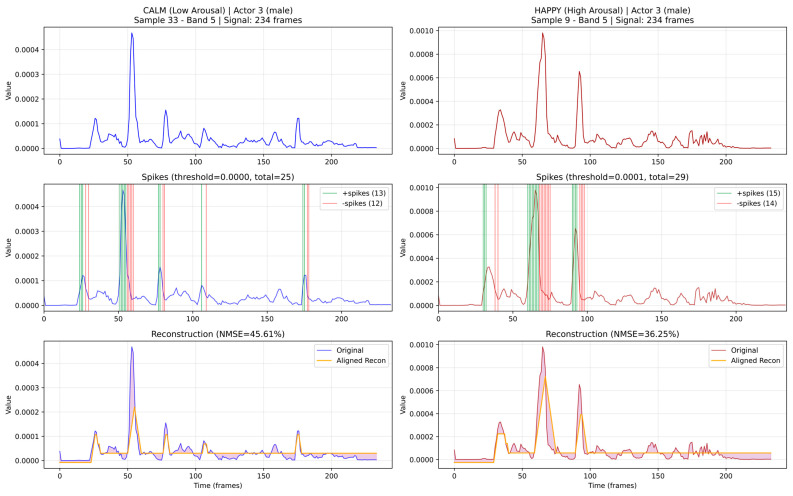
Example of linear-_fft features showing spike encoding and signal reconstruction. The spikes in green are positive and spikes in red are negative. The difference is presented as shadows. Spike encoding of linear FFT audio features (**top**) using the Step-Forward method (**middle**) and reconstructing the signals from the spikes back to the original ones (**bottom**).

The extracted audio features are then encoded into spikes using different possible encoding schemes. Figure 5 illustrates the encoding of the data from Figure 4 (top) using the Step-Forward method [17] (middle), as well as the reconstruction of the original signals from the spike trains (bottom), with the reconstruction error quantified by the normalized MSE. For a comparison, an example of 24 linear-fft feature extraction, encoding, and mapping, is given in Appendix A, Figure A1.

### 2.5. Tonotopic Mapping of Audio Features into a 3D SNNcube of the eXCube2 Framework

**Figure 6:**
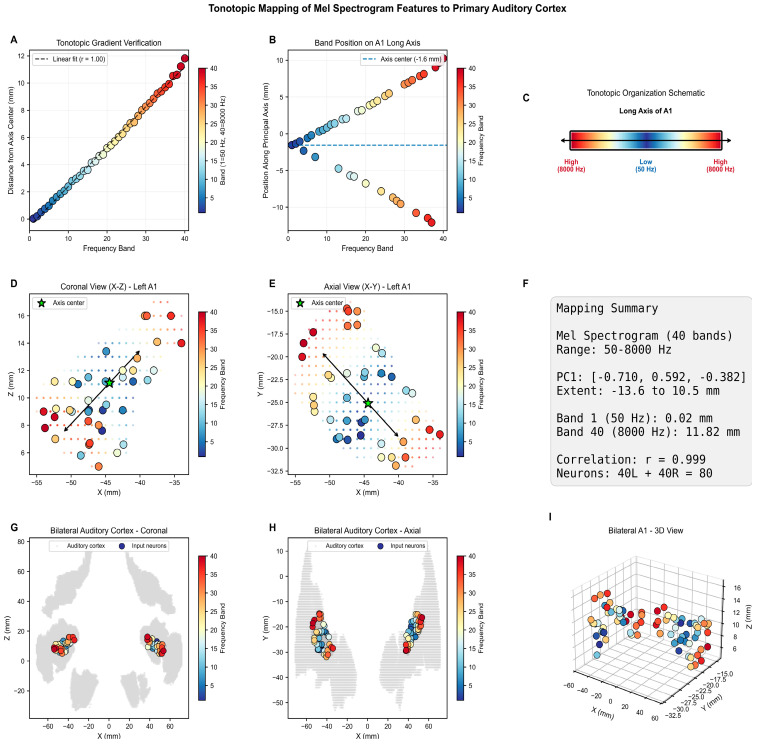
Mapping of mel-spectrogam audio features into a 3D SNNcube spatially structured according to the MNI brain template. PC1 = principle axis. (**A**) tonotpic gradient verification showing distance (mm) from axis center, (**B**) position of each mel band along the principal axis of A1, (**C**) frequency distribution along long axis, (**D**) coronal view, (**E**) axial view, (**F**) mapping summary, (**G**) bilateral coronal view, (**H**) bilateral axial view, (**I**) 3D view. rogam features into a 3D SNNcube spatially structured according to the MNI brain template. PC1 = principle axis.

**Figure A2:**
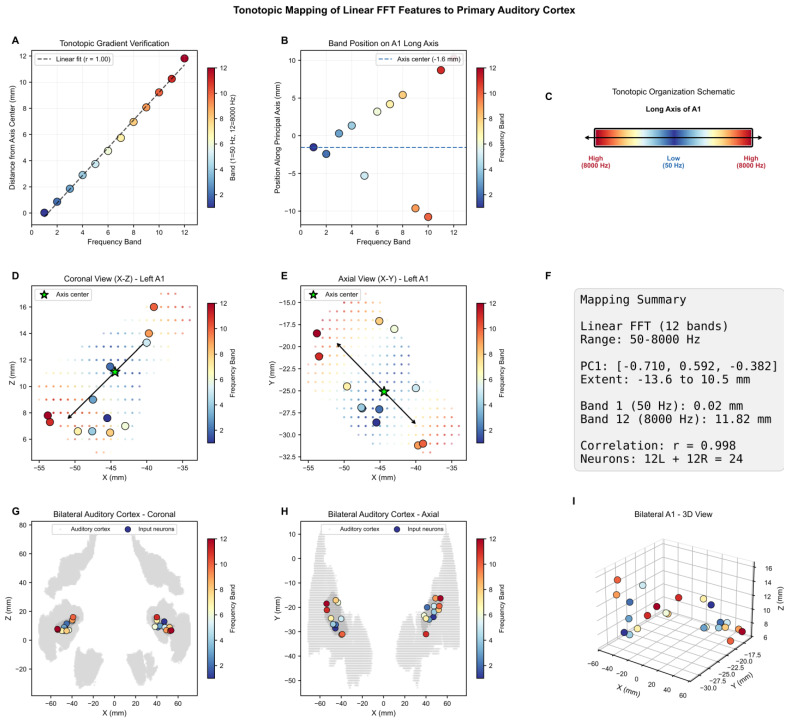
Mapping of linear-_fft features into a the SNNcube. PC1 = principle axis. (**A**) tonotopic gradient verification showing distance (mm) from axis center, (**B**) position of each mel band along the principal axis of A1, (**C**) frequency distribution along long axis, (**D**) coronal view, (**E**) axial view, (**F**) mapping summary, (**G**) bilateral coronal view, (**H**) bilateral axial view, (**I**) 3D view.

We employed a tonotopic mapping of audio features to the SNN reservoir, replicating the spatial organization of the human primary auditory cortex (A1), where neurons are arranged according to their preferred frequency. A1 follows a characteristic high → low → high frequency gradient along the long axis of Heschl’s gyrus: the cochlea unrolls frequency linearly, but the cortex folds this representation into two adjacent au-ditory fields (A1 and R) that meet at a low-frequency boundary, producing a mirror-symmetric gradient. This organization has been consistently demonstrated from early fMRI work [53], through high-resolution 7T studies confirming robust tonotopic gradient reversals centered on Heschl’s gyrus [54,55]. Following this principle, the extracted features are mapped into a pre-structured eXCube2 SNN using MNI brain template coordinates and a tonotopic assignment of spatial locations to the selected features. Figure 6 illustrates the mapping of the 40 × 2 = 80 mel-spectrogram features into the SNNcube, and the corresponding algorithm is presented in Table 2. For a comparative analysis, tonotopic mapping of linear-fft 24 audio features into the SNNcube is shown in Figure A2.

### 2.6. Visual Feature Extraction and Their Topographic Mapping in the eXCube2 Framework

Visual features are extracted from the RAVDESS video files as 52 facial blendshapes using MediaPipe Face Landmarker [50,51]. These comprise 5 brow features (browDownLeft/Right, browInnerUp, browOuterUpLeft/Right), 8 eye features (eyeBlinkLeft/Right, eyeSquintLeft/Right, eyeWideLeft/Right), 3 cheek features (cheekPuff, cheekSquintLeft/Right), 2 nose features (noseSneerLeft/Right), 4 jaw features (jawOpen, jawForward, jawLeft/Right), 28 mouth features (mouthSmileLeft/Right, mouthFrownLeft/Right, mouthPucker, mouthShrugUpper/Lower, among others), tongueOut, and a neutral baseline.

The 52 blendshape features are mapped bilaterally to the superior temporal sulcus (STS), which processes dynamic facial aspects including expressions, gaze, and speech movements [56]. Although STS-based face processing has often been characterized as right-hemisphere dominant, large-sample fMRI evidence shows this lateralization is weak, with only half of subjects showing clear right dominance [57], and TMS confirms that both left and right STS contribute causally to expression recognition [58]. The right STS exhibits clearer functional segregation between gaze, expression, and speech regions, while the left STS shows more distributed representations of the same movements [59], suggesting complementary rather than redundant hemispheric contributions.

Within each hemisphere, features are mapped topographically following the dorsoventral organization of STS [59]: dorsal regions (higher Z coordinates) encode upper-face features (brow, eye, and nose movements) while ventral regions (lower Z) encode lower-face features (mouth and jaw movements). The mapping occupies coordinates X = 28–60 mm (lateral), Y = −62 to −42 mm (posterior temporal), and Z = −11 to 16 mm (ventral to mid-level).

**Figure 7:**
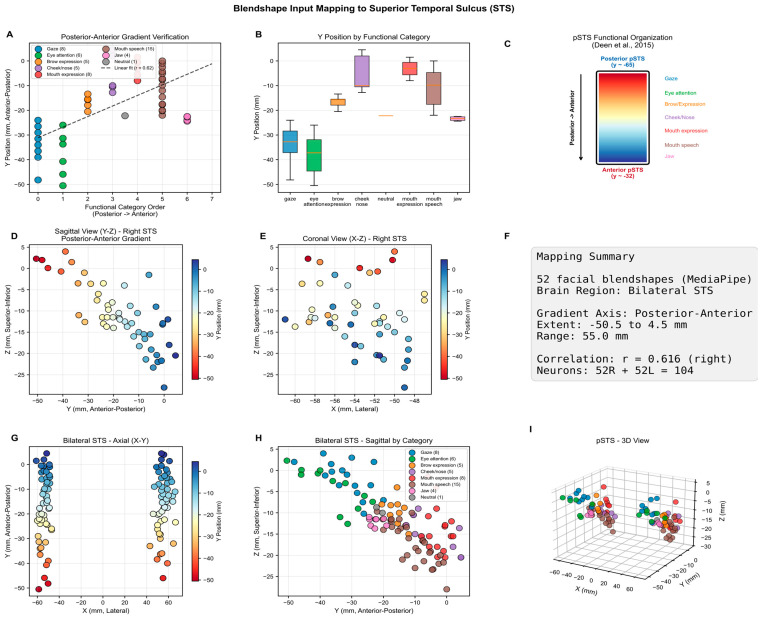
Mapping of blendshape visual features into a 3D SNNcube spatially structured according to the MNI brain template. (**A**) posterior-anterior gradient, (**B**) Y position by facial part and function, (**C**) functional gradient, (**D**) Sagittal view, (**E**) coronal view, (**F**) mapping summary, (**G**) bilateral axial view, (**H**) bilateral sagittal view, (**I**) 3D view.

This topographic mapping of the visual features is shown in Figure 7.

### 2.7. Mapping Multimodal Audio–Visual Data into an eXCube2 Model

**Figure 8:**
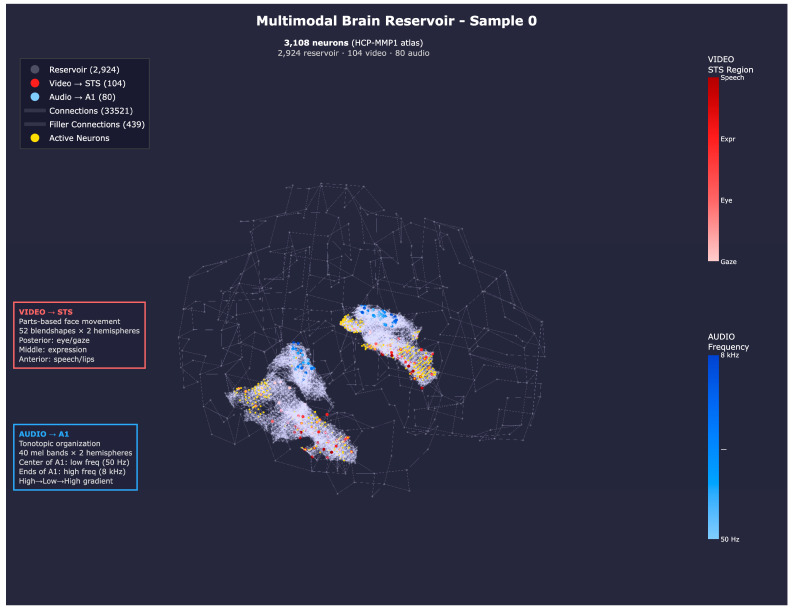
Propagated activity from audio input neurons (blue) and visual input neurons (red) for an exemplar multimodal sample (sample 0 from the benchmark dataset). Connections are densest within A1 and STS, reflecting the input mapping regions, though the recurrent reservoir architecture allows for activity to propagate across the entire brain volume through filler connections.

The audio and visual features described in the previous sections are combined to in the design of a multimodal audio–visual eXCube2 system. The extraction of audio and visual features is synchronized at 10 ms. A snapshot of the resulting feature activity for an exemplar multimodal data sample is shown in Figure 8.

### 2.8. Training of eXCube2 Models on Audio, Visual and Audio–Visual Data

Separate eXCube2 models are constructed for audio-only, visual-only, and multimodal audio–visual data using the features described above. For unsupervised training of the SNNcube, Spike-Timing-Dependent Plasticity (STDP) is employed (see [17]), with the training and testing parameters summarized in Table 3. Further experimental details and parameter settings for the training and testing procedures are provided in Appendix A.3.

After training each SNNcube model, state vectors are extracted from the reservoir and used to train a classifier in a supervised mode.

### 2.9. State Vector Extraction from a Trained SNNcube and Their Classification

Different approaches can be used to extract state vector from a trained SNNcube.

(a) Spike Count: This method sums the total number of spikes per neuron across all timesteps for each sample:
  (2) $$s_{i}=\sum_{t=1}^{T}\;x_{i}\left(t\right),$$
  where $\;x_{i}\left(t\right)$ denotes the spike activity of neuron *i* at time *t*. In this case, temporal information is aggregated into a single value per neuron, and the state vector is represented by the spike counts of all neurons.
(b) DeSNN weight-based state vectors: Alternatively, state vectors can be derived using the DeSNN encoding rule (see [47]), which computes a scalar value for each neuron based on its spiking activity. Specifically, each neuron’s value is determined by two properties of its spike train: the timing of its first spike (via rank-order coding) and its total spike count (via a drift component):
  (3) $$\omega_{i}=\alpha \;*\;m^{t_{i}^{first}}+d_{up}*\;\;n_{i}^{total}-d_{down}*\;\;\left(T-n_{i}^{total}\right),$$
  where α = 5.0, m = 0.8, $d_{up}$ = 0.8, $d_{down}$ = 0.01.

The collection of these values across all reservoir neurons forms the state vector for a given sample. The extracted state vectors are used to train a classifier to recognize two emotional states as two classes, corresponding to arousal and calm. In practice, simple spike counting performs comparably to DeSNN encoding because the reservoir has already transformed temporal information into spatial patterns. Through recurrent dynamics and STDP learning, different neurons become selective to different temporal motifs, and their firing patterns implicitly encode the temporal evolution of the input. Moreover, STDP strengthens connections between neurons that fire in consistent sequences, thereby em-bedding temporal structure into the network connectivity. Spike counting on reservoir neurons captures discriminative information, because each neuron’s firing reflects inte-grated temporal patterns across the network through learned connectivity. The reservoir performs temporal feature extraction.

Once the state vectors are extracted from the trained SNNcube, different classification methods have been applied and compared to classify these vectors into the two output classes as described in Table 4.

In the current implementation of the eXCube2 framework, the Learned Prototype classifier is used, as its clustering capability is well suited to the experimental data. Other classifiers can be employed for different applications while still using the same eXCube2 framework.

## 3. Experimental Results, Interpretability and Explainability of the eXCube2 Models

### 3.1. Classification Results on the Experimental Data

Table 5 compares classification performance obtained using three eXCube2 models for: (1) multimodal data; (2) audio data only; (3) visual data only. The eXCube2 model can operate on the integrated multimodal input as well as on each modality separately. Using different methods for state vector extraction and classification yields comparable results, with accuracies consistently in the range of around 80%.

Legend:

▪ I = Input neurons only (spike-encoded features)
▪ R = Reservoir neurons only
▪ I + R = Combined input + Reservoir neurons
▪ SC = Spike count (sum of spikes over time)
▪ split = Separate positive/negative spike counts (doubles feature dimensionality)
▪ STS = Superior Temporal Sulcus brain region reservoir neurons only (audio–visual integration)
▪ A1 = Primary Auditory Cortex brain region reservoir neurons only
▪ py = DeSNN Python V1.0 implementation (from NeuCubePy library)
▪ hybrid = DeSNN connecxtion weights analysis.

Using the introduced “don’t know” output with a confidence threshold in the range 0.55–0.65 improves the effective classification accuracy to up to 89% by rejecting low-confidence samples, as shown in Table 6.

**Figure 9:**
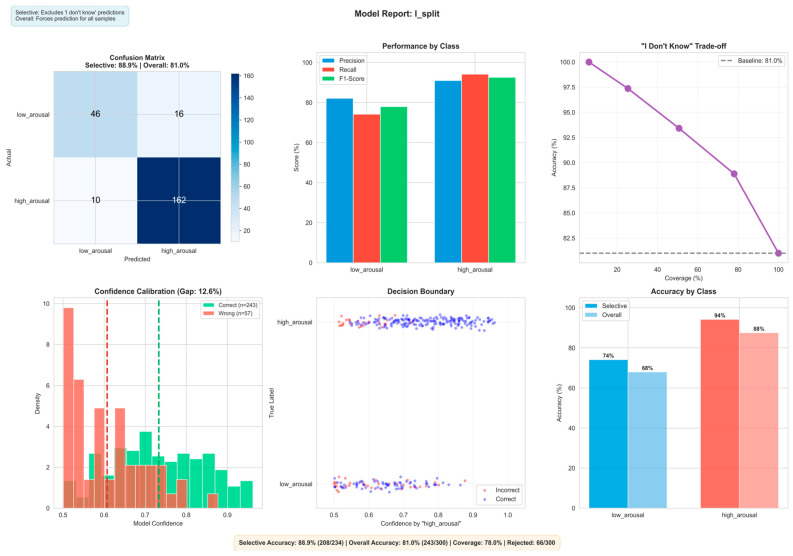
Visualisation of classification results on multimodal audio–-visual data using the multimodal eXCube2 model. Showing confusion matrix (**top-left**), per-class precision/recall/F1-score (**top-centre**), selective classification trade-off (**top-right**), confidence calibration showing the density of correct vs. incorrect predictions across confidence levels, where a lower gap indicates better-calibrated scores (**bottom-left**), per-sample confidence distribution with decision boundary (**bottom-centre**), and per-class accuracy (**bottom-right**).

**Figure 10:**
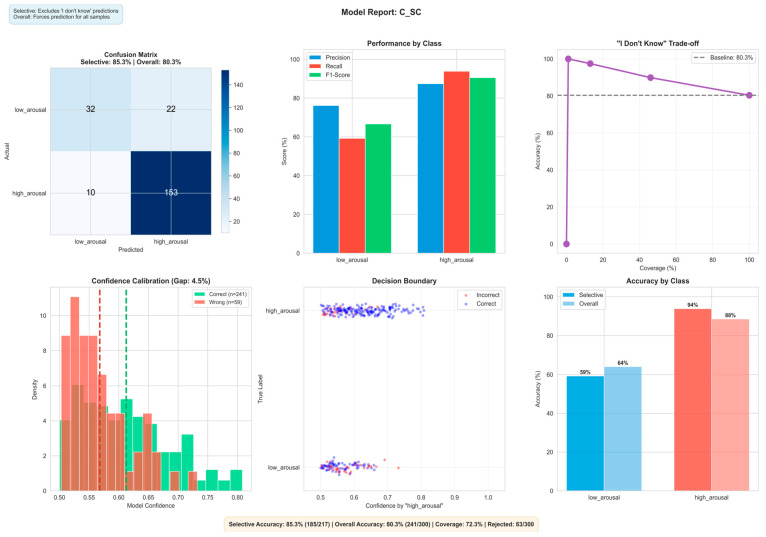
Visualisation of classification results on audio data using the audio eXCube2 model. Showing confusion matrix (**top-left**), per-class precision/recall/F1-score (**top-centre**), selective classification trade-off (**top-right**), confidence calibration showing the density of correct vs. incorrect predictions across confidence levels, where a lower gap indicates better-calibrated scores (**bottom-left**), per-sample confidence distribution with decision boundary (**bottom-centre**), and per-class accuracy (**bottom-right**).

**Figure 11:**
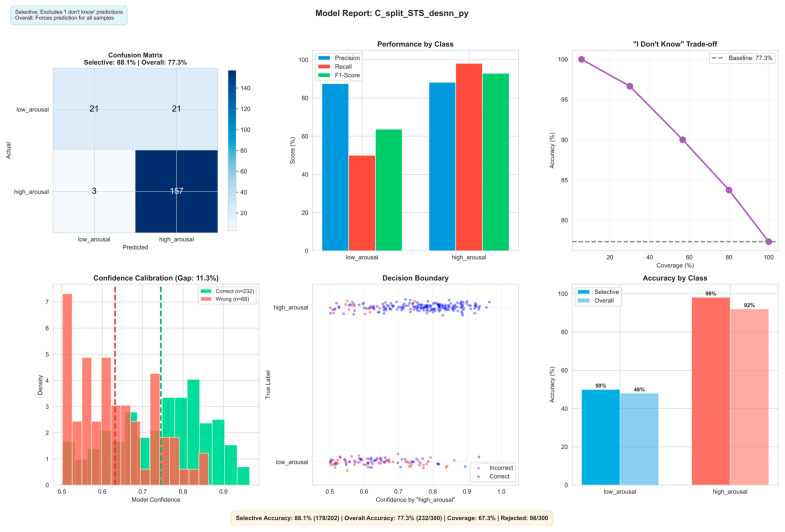
Visualisation of classification results on visual data using the visual eXCube2 model. Showing confusion matrix (**top-left**), per-class precision/recall/F1-score (**top-centre**), selective classification trade-off (**top-right**), confidence calibration showing the density of correct vs. incorrect predictions across confidence levels, where a lower gap indicates better-calibrated scores (**bottom-left**), per-sample confidence distribution with decision boundary (**bottom-centre**), and per-class accuracy (**bottom-right**).

More details of the three classification models and their interpretation are presented in Figure 9, Figure 10 and Figure 11, respectively, and in the next sub-section. They explain the best state vector extraction method and random seed initiated for the learned prototype according to accuracy.

### 3.2. Interpretation of the Classification Results

The multimodal model uses input neurons with their positive and negative spikes represented as two different features. The model achieves 88.9% selective accuracy using ‘I don’t know’ at a 78.0% coverage of the full data, and 81.0% accuracy on the full predictions. High arousal accuracies dominate at 88–94%, whereas low arousal accuracies range from 68–74%. This is also most likely due to class imbalance from the raw dataset; however the multimodal model bridges this discrepancy more than the other models. Where more data is available such as with the high arousal, we can see that the accuracy percentages reach the high 80 s/mid 90 s. The multimodal model also covers more of the full data, whilst reaching higher accuracies compared to the unimodal models.

The audio model uses a combined input and reservoir spike count, achieving 85.3% selective accuracy using “I don’t know” at a 72.3% coverage of the full data, and 80.3% accuracy on the full predictions. High arousal accuracies dominate at 88–94%, whereas low arousal accuracies range from 59–64%. This is most likely due to class imbalance from the raw dataset. The model performs slightly better than the video model on low arousal. Where more data is available such as with the high arousal, we can see that the accuracy percentages reach the high 80 s.

The video eXCube2 model uses a combined input and reservoir spike count, where inputs have their positive and negative spikes represented as two different features. This is concatenated with the deSNN_py state vector extracted from STS neurons in the reservoir, meaning only the relevant neurons related to visual processing are used. The model achieves 88.1% selective accuracy using “I don’t know” at a 67.3% coverage of the full data, and 77.3% accuracy on the full predictions. High arousal accuracies dominate at 92–96%, whereas low arousal accuracies range from 48–50%. This is also most likely due to class imbalance from the raw dataset. The model is much better at detecting high arousal versus the audio model, but worse off for low arousal. Where more data is available such as with the high arousal, we can see that the accuracy percentages reach the 90 s. The selective prediction mechanism “don’t know”, allows for the model to abstain from low-confidence predictions by thresholding the classifier’s softmax output.

Figure 9, Figure 10 and Figure 11 present the scope-accuracy tradeoff curves for all three models. At full coverage (100%), the multimodal model achieves 81.0% accuracy. As the confidence threshold increases, coverage decreases while accuracy improves, at 78% coverage, accuracy reaches 88.9%. This tradeoff is continuous and configurable at deployment time without retraining.

The abstention capability has direct practical utility. In client-facing applications or streaming scenarios where data is readily available, the system can request additional information when uncertain and defer prediction until confidence is sufficient. This is analogous to a clinician requesting further tests before diagnosis. While experimental evaluation requires forced prediction on a fixed test set, in deployed systems the “don’t know” response enables the model to maintain high reliability on the predictions it does make, which is preferable to forcing unreliable predictions.

In terms of scalability, the models offer several inherent advantages. Learning is local through STDP and DeSNN state vectors, eliminating the need for backpropagation through time and the associated memory overhead. Computation is also inherently sparse, as the spiking reservoir only processes activity when spikes occur rather than performing dense matrix operations at every timestep. The architecture is also modular, as additional brain regions, data modalities, or feature types can be incorporated by mapping new inputs to their corresponding cortical areas within a single unified reservoir. Because all regions share the same recurrent dynamics, activity in one area influences processing in another as it unfolds in time, enabling genuine cross-modal interaction rather than independent parallel streams combined after the fact.

Furthermore, the spiking architecture is well-suited for future deployment on neuromorphic chips, which could offer significant gains in energy efficiency and processing speed through hardware-level spike routing. However, in its current software implementation the model is sequential on two levels: within each sample, the reservoir must process timesteps in order as the activity at each step depends on all preceding steps, and across samples, the incremental learning procedure updates the reservoir after each training instance, meaning samples cannot be processed in parallel. One potential mitigation is training multiple reservoirs independently and averaging their learned weights, though how effectively such ensemble strategies translate to spiking architectures remains an open question.

Reproducibility: All classification results are reported as means with 95% confidence intervals computed over 30 independent random seeds. The narrow confidence intervals (typically ±0.1–0.2 percentage points) confirm high stability across classifier initializations. For example, the multimodal model achieves 82.1% [95% CI: 82.0, 82.3] overall accuracy.

Computational Cost: Table 7 reports the computational cost of each pipeline stage for a data length 3–5 s, measured on a single CPU core (Apple M-series, no GPU required).

End-to-end inference time from raw video to emotion prediction is approximately 2.1 s, dominated by the reservoir forward pass (1.0 s) and feature extraction (1.0 s). Total training time for the full pipeline is approximately 49 min for 1520 samples. The SNN reservoir contains 3108 LIF neurons with a sparse connectivity matrix 27,741–45,214 non-zero connections (0.3–0.5% density), requiring <1 MB in sparse format. The complete trained model (reservoir weights + classifier) occupies <40 MB.

For comparative analysis, Appendix B, Table A1, shows classification using typical machine learning methods on feature vectors, directly extracted from raw data, rather than from the SNNcube of an eXCube2 model. The experiments demonstrate that using state vector features extracted from a trained SNNcube in the eXCube2 model lead to a much higher accuracy, along with interpretability, explainability and adaptability of the models, when compared with the accuracy of models than use features extracted directly from raw data.

The proposed eXCube2 framework allows for the system to be used by new speakers in an on-line interactive mode, rather than tested by recorded speakers from the RAVDESS benchmark data set. Examples of using the system by an English speaking male of Chinese origin and a female of European origin are given in Table A2 and Table A3 of Appendix B.

### 3.3. Explainability of the eXCube2 Models

The eXCube2 models provide informative representations at multiple levels of their structure and dynamics. First, the spatial mapping of features is itself interpretable, as it follows a brain template and incorporates established neuroscience knowledge (e.g., Figure 6, Figure 7 and Figure A1).

**Figure 12:**
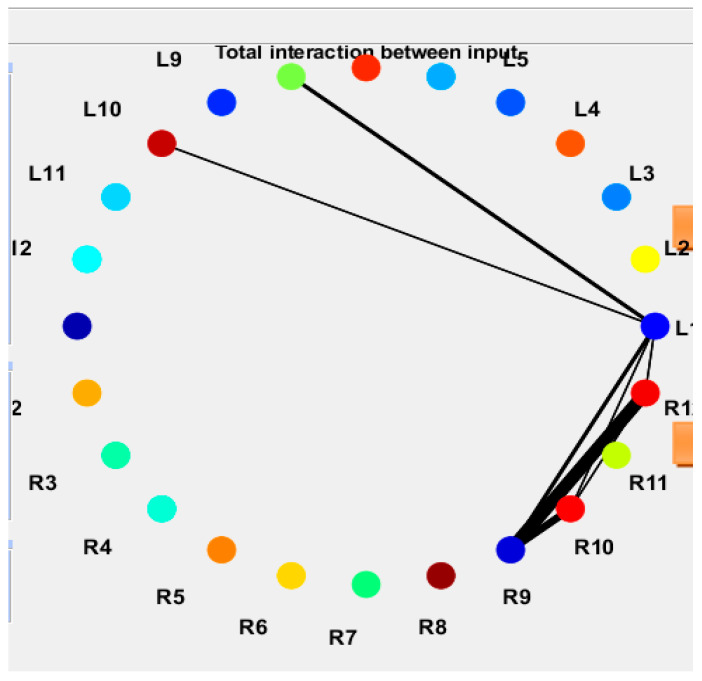
Spike–time association between 24 linear-fft features (see Appendix A.1 for their mapping into the SNNcube), shown as arcs between features represented as nodes (L means left hemisphere; R means right hemisphere). Thicker arcs indicate a higher number of spikes at the next time step (10 ms) at a node following a spike in its connected node, representing information exchange in spike form.

More importantly, the models capture dynamic interactions between features. This is illustrated in Figure 12, which shows spike-time associations between 24 linear-fft features (see Appendix A.1 for their mapping into the SNNcube). The learned associations are visualized as arcs between features represented as nodes (L denotes the left hemisphere; R denotes the right hemisphere). The thicker the arc, the more frequently spikes at one node are followed by spikes at the connected node in the next time step (10 ms), indicating stronger temporal coupling. This provides a spike-based representation of information exchange within the model.

**Figure 13:**
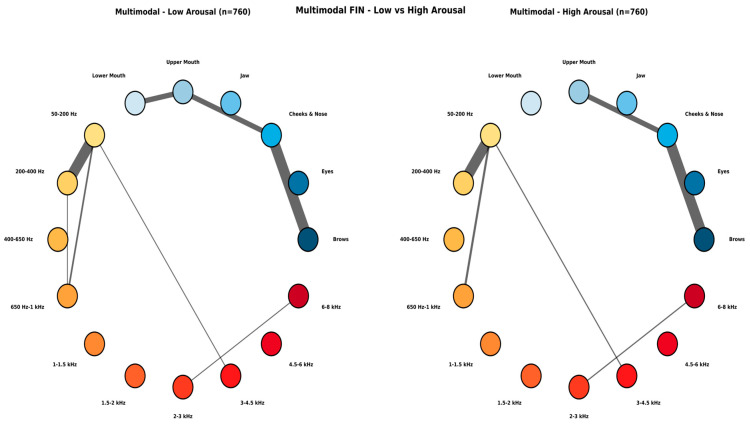
Feature Interaction Network (FIN) for the multimodal model, comparing low arousal (**left**) and high arousal (**right**) classes. Nodes represent six anatomical face regions (in blue) and ten mel-spectrogram frequency groups (in red), aggregated from 184 input features (L + R combined). Line thickness indicates the strength of spike–time association between feature groups.

Figure 13 shows feature interaction between most prominent audio and visual features of the audio-visual eXCube2 model when mel-spectrogram audio features are used along with the face features. In both classes of emotional states, audio–audio interactions dominate, concentrated among low-frequency bands (50 Hz to 1 kHz), with no notable differences in cross-modal connections emerging. In the low arousal condition, the connection between 50–200 Hz and 200–400 Hz is stronger, an additional link appears between 200 and 400 Hz and between 650 Hz and 1 kHz, and the upper and lower mouth regions are connected. In the high arousal condition, these three connections weaken or disappear, suggesting a decoupling of both the low-frequency audio bands and the mouth regions.

## 4. Conclusions, Discussions, Limitations and Future Work

The paper presents a novel SNN-based framework eXCube2 for the recognition and classification of emotional states from audio, visual or combined audio–visual data. The framework consists of modules for feature extraction, spike encoding, mapping features into a 3D SNN, pre-structured according to a brain template, training the SNNcube, extracting state vectors from the SNNcube, training a classifier on the state vectors, recalling and adapting the system to new data, as well as visualisation and dynamic explanation of the processing. Each module is grounded in brain-inspired information processing principles.

We have used a benchmark data set to illustrate our new approach, but the goal of the paper is not to achieve perfect statistical validation results on the benchmark RAVDESS data set through cross-validation. In Appendix B new experimental results on the same data with cross validation tests are shown when feature vectors are extracted from the raw data rather than from the SNNcube (Table A1). The goal is for the framework to be further adapted and tested on new speakers of different accents, pronunciations and culture-based expression. In Appendix B we have tested the framework on a new male speaker of Chinese origin (Table A2) and a female speaker of European origin (Table A3) with satisfactory results. The framework allows for further testing and adaptation on new speakers.

Several limitations of the current study should be noted. The eXCube2 models address the problem of binary arousal classification (high vs. low). Addressing the problem of full eight-emotion taxonomy available in RAVDESS is another problem that can follow. The RAVDESS dataset, while well-established, consists of acted emotional expressions from 24 North American English speakers. Generalization to spontaneous emotions, diverse languages, and cross-corpus settings remains to be demonstrated. The acoustic features used for data visualisation in this study, including RMS energy, spectral centroid, spectral flux, and low-frequency energy, are associated with emotional dimensions at the population level. However, these relationships are not deterministic. Individual variation in vocal expression, cultural differences, and speaker-specific characteristics mean that the same emotion may manifest with different acoustic profiles across speakers and contexts. The visualisations presented in this work should therefore be interpreted as illustrative of general trends rather than universal patterns.

The current model uses a 3108-neuron reservoir, which serves as a proof-of-concept. The software architecture supports scaling to substantially larger reservoirs, which may enable finer-grained temporal pattern capture. Class balancing in the training set was achieved through sample duplication rather than data augmentation techniques (e.g., pitch shifting, noise injection), which may limit the diversity of learned representations for the minority class.

At each stage of the framework, multiple methods can be employed, some of which were explored in this work. An integrated eXCube2 system has been developed for audio–visual data, combining three models for audio-only, visual-only, and multimodal processing. The system was evaluated on benchmark datasets for each modality and their integration, achieving accuracies of up to around 89% when confidence-based rejection is applied.

Future work can involve development of eXCube2 models of audio-visual data for other problems rather than emotional state recognition. SNN models have already been used for mental state assessment based on audio–visual data. The work [30] proposes a Transformer-based multimodal mental health assessment framework that models audio–visual–text cues from interview sessions using a cross-attention fusion mechanism to capture inter-modal dependencies relevant to distress and mood assessment. The approach uses strong pretrained feature extractors for each modality—wav2vec 2.0 for audio, ResNet-50 for facial/visual features, and BERT for text embeddings—followed by modality-specific Transformer encoders to learn intra-modality temporal/contextual structure [30]. The central methodological contribution is a Cross-Attention Transformer Block that performs dynamic fusion: for each target modality X∈{A,V,T}, the model computes cross-attention using X as the query and the concatenation of the other modalities as keys/values, enabling the system to selectively attend to complementary signals conditioned on task relevance. This design is explicitly positioned as an improvement over static concatenation/early fusion, with added interpretability through attention-weight visualization. The evaluation uses two publicly available datasets: the Bipolar Disorder Corpus and the Extended Distress Analysis Interview Corpus (E-DAIC), both consisting of audio–visual interview recordings with corresponding text transcriptions and mental-health/emotion-related labels. Preprocessing includes the following: audio resampled to 16 kHz and normalized; video keyframes extracted at 1 fps, with face detection/alignment using MTCNN and resized to 224 × 224; text tokenized with the BERT tokenizer and padded to fixed length [30].

Another application area of SNN is targeting moving object recognition. Paper [31] presents a Transformer-based Spiking Neural Network (SNN) for multimodal audio–visual classification, targeting accurate fusion under SNN constraints while emphasizing efficiency. The proposed model, termed SMMT, integrates unimodal spiking backbones with a Spiking Cross-Attention (SCA) module that performs bidirectional audio↔vision interaction and incorporates relative position bias and dropout to improve temporal/positional modelling and generalization. Audio is represented via log-mel spectrograms (STFT-based), consistent with standard environmental sound processing [31].

The above examples point to a future use of the eXCube2 framework in a wider range of domains using one or both audio–visual modalities, including: monitoring response to treatment over time [14]; evaluation of user satisfaction in online services [61]; human–robot interaction [62]; chatbots [28]; interactive games, and related applications.

The three models are implemented as Python software tools (V1.0), enabling the design and experimentation of eXCube2 models across sound, image, and video data. The use of an SNNcube together with evolving classifiers supports scalability to larger datasets, adaptability to new data, and explainability of the underlying dynamic processes. A software module is developed to dynamically visualize and explain the activity of eXCube2 during recall on new data.

Importantly, the SNN-based implementation of Brain-Inspired AI (BIAI) makes the framework suitable for deployment on neuromorphic hardware platforms, enabling reduced power consumption, smaller device size, and improved efficiency, while preserving adaptability and explainability [63,64,65,66,67]. Ultimately, the use of shared brain templates for both biological and artificial systems may help bring human–machine symbiosis closer in the future.

Future work will focus on: (1) evaluating additional classifiers for the extracted state vectors (e.g., [68,69]); (2) in addition to audio–visual data, using brain data (e.g., EEG, fMRI) and other data modalities to integrate in an eXCube2 model [48,70]; (3) further extending the concept of evolving spatio-temporal associative memory based on brain principles [39,71]; and (4) implementing the models on contemporary software and hardware platforms for real-world applications [17,66,72,73]. The proposed framework can be integrated with Transformer-based systems [74,75,76,77,78,79,80,81,82,83,84] making them brain-inspired and more efficient. It also extends the theoretical studies on emotion recognition methods [85,86] and the methods for abnormal brain state diagnosis (e.g., Parkinson’s disease, Alzheimer’s disease, ADHD, dementia) [16,87].

## Figures and Tables

**Table 1:** Audio features considered in this study.

Features	Number	Frequencies	In Both Sides	Previous Usage
Mel-spectrogram	40	50–8000 Hz (mel)	80	State of the art emotion recognition
Mel-fft	12	50–8000 Hz (mel)	24	Biologically plausible
Linear-fft	12	50–8000 Hz (linear)	24	Technical analysis
mfcc	12	Cepstral coefficients	24	Speaker-independent speech recognition

**Table 2:** Algorithm for downsampling and mapping audio features into the SNNcube.

For each hemisphere, the following was carried out: Extract full-resolution A1 coordinates Identify downsampled neurons within A1 Apply PCA to estimate the principal axis of A1 (tonotopic gradient direction) Project neurons onto this axis to obtain a normalised tonotopic position in [0, 1] Select neurons evenly spaced along the gradient to match the number of frequency bands Map each audio feature column to one A1 neuron: - Left channel (columns 1 to N) → Left A1 neurons - Right channel (columns N + 1 to 2N) → Right A1 neurons Neurons are ordered by tonotopic position, so: - Band 1 (lowest frequency, ≈50 Hz) → maps to the center of A1 - Band N (highest frequency, ≈8000 Hz) → maps to the ends of A1 Perform direct mapping where feature column i → neuron i (1-indexed bands), as summarized below:		
Method	Left Channel Mapping	Right Channel Mapping
Mel-spectrogram	bands 1–40 → neurons 1–40	bands 1–40 → neurons 41–80
Mel-fft	bands 1–12 → neurons 1–12	bands 1–12 → neurons 13–24
Linear-fft	bands 1–12 → neurons 1–12	bands 1–12 → neurons 13–24
mfcc	bands 1–12 → neurons 1–12	bands 1–12 → neurons 13–24

**Table 3:** Audio and visual features and parameters.

Feature	Audio	Visual
Features	80 mel_spectrogram	104 facial blendshapes
Brain region	Bilateral auditory cortex (A1)	Bilateral STS
Input map	Tonotopic (low → high freq)	Topographic
Reservoir	3108 neurons	3108 neurons
Train samples	1520	1520
Test samples	300	300

**Table 4:** The used classification methods for the classification of state vectors.

Method	Mathematical Formulation	Description
(a) SVM (RBF Kernel)	$K\left(x_{i},\;x_{j}\right)=exp\left(-\gamma \;"."\;{\left|\left|x_{i}-x_{j}\right|\right|}^{2}\right)$	Gamma set automatically. Maximum-margin hyperplane in kernel space.
(b) Weighted Weighted KNN (WWKNN) [60]	$d\left(x,\;x^{\prime }\right)=\sqrt{\underset{f}{\sum }SNR_{f}{\left(x_{f}-{x^{\prime }}_{f}\right)}^{2}}$	Feature-wise SNR weighting, where SNR_f = variance_between(f)/variance_within(f). Downweights noisy features, emphasises discriminative ones.
(c) Centroid Prototype	$p_{c}=\frac{mean\left(x_{c}\right)\;}{\left|\left|mean\left(x_{c}\right)\right|\right|}$	Class represented by a normalised centroid; classification by maximum cosine similarity.
(d) Learned Prototype	$L=-\underset{i}{\sum }log\;\frac{exp\left(sim\left(x_{i},\;p_{y_{i}}\right)\;/\;tau\right)}{\sum_{c}exp\left(sim\left(x_{i},\;p_{y_{i}}\right)\;/\;tau\right)}$	Prototypes optimised via gradient descent on cross-entropy loss. Adam optimiser, lr = 0.01, 300 epochs, tau = 0.1.

**Table 5:** Accuracy of the three models developed in this study, along with the top 3 methods used for the state vector extraction, using the best random starting seeds out of 30 for the learned prototype classifier.

eXCube2 Model	Method	Mean Acc ± Std	95% CI
Multimodal	I_split	82.1% ± 0.4%	[81.95%, 82.25%]
	STS_desnn_py	81.9% ± 0.2%	[81.82%, 81.94%]
	desnn_hybrid	81.3% ± 0.5%	[81.11%, 81.46%]
Audio		80.2% ± 0.3%	[80.07%, 80.26%]
		79.9% ± 0.3%	[79.79%, 80.01%]
		78.6% ± 0.4%	[78.45%, 78.75%]
Video		80.5% ± 0.2%	[80.48%, 80.61%]
		80.5% ± 0.2%	[80.48%, 80.61%]
		80.5% ± 0.3%	[80.38%, 80.62%]

**Table 6:** Comparative best seed accuracy of the eXCube2 models on audio, visual and audio–visual data when a “don’t know” output is introduced.

Model	Confidence Threshold	Coverage	Overall Accuracy	Selective Accuracy	Correct	Wrong	Rejected	Lost Correct	Errors Avoided
Video	65%	67.3%	77.3%	88.1%	178	24	98	54	44
Audio	55%	72.3%	80.3%	85.3%	185	32	83	56	27
Multimodal	60%	78.0%	81.0%	88.9%	208	26	66	35	31

**Table 7:** Computational cost of each pipeline stage, measured on a single CPU core (Apple M-series silicon, no GPU). Training times are for the full RAVDESS dataset (1520 samples, ~3–5 s video clips). Inference time is end-to-end from raw video input to emotion prediction. Feature extraction is performed once offline during training but included in the inference path for deployment-realistic timing.

	Per Sample	Training (1520)	Inference (1)
Feature extraction (mel)	175 ms	4.5 min	175 ms
Feature extraction (blendshapes)	800 ms	20 min	800 ms
Spike encoding	0.2 ms	<1 s	0.2 ms
Reservoir simulation + STDP	871 ms	22 min	—
Reservoir forward pass (no STDP)	—	—	1025 ms
State vector extraction (DeSNN)	81 ms	2.1 min	81 ms
Classifier training (300 epochs)	—	~30 s	—
Classifier prediction	<1 ms	—	<1 ms
Total		~49 min	~2.1 s

## Data Availability

The Python implementation of the eXCube2 framework and the three models presented in this paper are available upon request, subject to copyright restrictions (contacts: T.L. and A.Y.). The NeuCube Python implementation (NeuCubePy) is available online at https://github.com/KEDRI-AUT/NeuCube-Py, V1.0 (accessed 30 November 2025).

## Appendix A. Technical Implementation Details

### Appendix A.2. Experimental Details of the Tonotopic Mapping of Audio Features into the SNNcube

The following setup was used for the tonotopic mapping of audio features:

- The HCP-MMP1 atlas [44] on MNI152 template was used.
- A hybrid downsampling scheme was applied: 2.5 mm resolution for the auditory cortex and 8.1 mm for the rest of the brain, resulting in 4176 neurons in total (840 auditory + 3336 other neurons).
- Input neurons were placed only in A1 (primary auditory cortex) in both hemispheres.
- A PCA-based gradient was used to distribute neurons evenly along the tonotopic axis (high → low → high frequencies).
- A direct 1:1 mapping was applied: sample column i → neuron i

The HCP-MMP1 atlas provides precise anatomical boundaries for auditory regions:

- A1 (Core): Primary auditory cortex with sharp frequency tuning and direct sensory input.
- Belt: Surrounding A1, integrates frequency channels and supports phonemes/timbre processing
- Parabelt: Higher-level auditory processing, including speech and music categories.

Features were mapped specifically to the A1 region of the SNNcube for the following reasons:

- Biological plausibility: Mimics how the real auditory cortex receives input.
- Spatial learning: Enables the SNN to learn spatial relationships between frequency bands.
- Interpretability: Neuron activations correspond to known brain regions.
- Emergent organization: As shown in TopoAudio (29), spatially constrained networks can develop brain-like organization without explicit supervision.

With this mapping, the following behaviour is observed:

- Only A1 (core) receives direct input, not belt or parabelt regions.
- A1 neurons are frequency-selective, similar to mel spectrogram or fft bands.
- Belt regions receive processed output from A1 via learned SNN connections.
- Parabelt regions receive output from belt regions.
- This mapping matches the biological processing hierarchy.

From the HCP-MMP1 atlas on MNI152 template, the hybrid downsampling is applied as follows:

- Auditory cortex: 2.5 mm voxel size (high resolution for input neurons).
- Other regions: 8.1 mm voxel size (standard NeuCube resolution).
- Result: 840 auditory neurons + 3336 other neurons = 4176 total neurons.

### Appendix A.3. Experimental Details of the Training/Testing Parameters of the eXCube2 Models

The following parameters were used for the three experiments: audio-only, visual-only, and multimodal audio–visual:

Initial dataset (before oversampling):

- Training: 360 samples (6 actors × 60 recordings)
- Testing: 120 samples (2 actors × 60 recordings)

Class imbalance in training data:

- Low arousal (neutral, calm, sad): 120 samples
- High arousal (happy, angry, fearful, disgust, surprised): 240 samples
- Ratio: 1:2 (imbalanced)

After oversampling (training only):

- Low arousal: 240 samples (duplicated from 120)
- High arousal: 240 samples (unchanged)
- Total training set: 480 samples (balanced 1:1)

Test set (unchanged distribution):

- 40 low arousal + 80 high arousal = 120 samples

Signal duration:

- 234–501 timesteps after silence trimming
- Shortest: 234 timesteps × 10 ms = 2.34 s
- Longest: 501 timesteps × 10 ms = 5.01 s

Feature extraction parameters:

- 10 ms hop size, 25 ms window length (standard in speech processing)

Full dataset split:

- Training: 1520 samples (760 class 0, 760 class 1), 19 actors (3–12, 15–23)
- Testing: 1820 samples (860 class 0, 960 class 1), 5 actors (1, 2, 13, 14, 24) Appendix B. Baseline Experiments: Classification of Audio Feature Vectors Directly Extracted from Raw Data

## Appendix B. Comparative Testing Results

### Appendix B.1. Experiments on State Feature Vectors Directly Extracted from Raw Data Using Machine Learning Methods

To provide an additional quantitative justification for the use of the brain-inspired eXCube2 models, here we provide a baseline experiment, conducted on audio feature vectors extracted directly from raw data, in contrast to the state vectors extracted from the SNNcube in the eXCube2 models. The dataset consists of 480 training samples and 120 testing samples, each represented as 80 mel_spectrogram feature vectors. All input features were standardized prior to training. The 480 training samples correspond to approximately 31% of the 1520 training samples used in the full eXCube2 temporal experiments.

Multiple baseline classifiers were evaluated. Each model was trained and tested several times to ensure stability and to explore performance improvements through controlled increases in model capacity. The following classifiers were examined using the NeuCom software, Student version V0.919 [88]:

- Support Vector Machine (SVM) with linear kernel;
- SVM with polynomial kernels (degrees 2, 3, and 4);
- SVM with RBF kernel;
- Multilayer Perceptron (MLP) with linear activation;
- Multilayer Perceptron (MLP) with nonlinear activation;
- Evolving Classification Functions (ECF).

Model capacity was progressively increased (e.g., higher polynomial degrees, larger hidden layers). In several configurations, near-perfect training accuracy was achieved. However, these cases consistently exhibited overfitting, with limited improvement in test accuracy. Increasing model complexity did not lead to substantial gains in generalization performance. Classification results are shown in Table A1.

**Table A1:** Classification test accuracy on audio feature vectors directly extracted from raw data.

Model	Test Accuracy
SVM Linear	64.2%
SVM Polynomial (deg2)	70.8%
SVM Polynomial (deg3)	67.5%
SVM Polynomial (deg4)	73.3%
SVM RBF	66.7%
MLP (linear)	~62.0%
MLP (non-linear)	~69.0%
ECF	71.7%

Across all configurations, test accuracy saturated within the range of approximately 60–74%. Although higher-capacity models achieved very high training performance, generalization performance remained limited within a narrow range.

This experiment demonstrates that using state vector features extracted from a trained SNNcube in the eXCube2 model, as shown in Section 3 of the paper, achieving 80+ accuracy along with interpretability, explainability and adaptability of the models, is superior in several aspects than using features extracted directly from raw data.

### Appendix B.2. Testing eXCube2 Models on New Speakers

The proposed eXCube2 framework and the developed software allow for the system to be used by new speakers in an on-line interactive mode, rather than tested by recorded speakers from the RAVDESS benchmark data set. Examples of using the system by an English speaking male of Chinese origin and a female of European origin are given in Table A2 and Table A3 below.

**Table A2:** Classification accuracy of emotional state recognition of an English speaking male of Chinese origin, when using the three eXCube2 models trained on the RAVDESS data of American subjects. The highest correct classification accuracy achieved by a model is highlighted in bold.

Input Data/Model Type	Model Output Aroused	Model Output Calm	Confirm Threshold	Prediction
*eXCube2 for audio data*				
Speech input—Aroused	**68.5%**	31.5%	55%	**Class 1**
Speech input—Calm	41.7%	**58.7%**	55%	**Class 0**
*eXCube2 for visual data*				
Face input—Aroused	**72.2%**	27.8%	55%	**Class 1**
Face input—Calm	40%	**60%**	55%	**Class 0**
*eXCube2 for audio–visual*				
Multimod. input—Aroused	**75.4%**	24.6%	55%	**Class 1**
Multimodal input—Calm	33.6%	**66.4%**	55%	**Class 0**

**Table A3:** Classification accuracy of emotional state recognition of an English speaking female of European origin, when using the audio eXCube2 model, trained on the RAVDESS data of American subjects. The highest correct classification accuracy achieved by a model is highlighted in bold. This shows the need to optimize the confirmation threshold for speakers of different origins.

Input Data/Female Europe Model Type: Audio eXCube2	Model Output Aroused	Model Output Calm	Confirm Threshold	Prediction
Speech input—Aroused	50.4%	49.6%	55%	Don’t know
Speech input—Calm	44.9%	**55.1%**	55%	**Class 0**

If new data are misclassified, the framework can be incrementally trained and adapted to the new data as this is a prominent feature of the used brain-inspired SNN architecture [17,18]. The framework can also be trained on different data sets and the use of the RVDNESS data are just one example.
